# Mitochondrial micro RNAs: Crucial players in carcinogenesis

**DOI:** 10.32604/or.2025.055945

**Published:** 2025-05-29

**Authors:** ASHUTOSH KUMAR MAURYA, ANJALI SANGEETH, RABINA PUNATHIL, R. GRACE RAJI, V.B. SAMEER KUMAR

**Affiliations:** 1 Department of Biochemistry & Molecular Biology, Central University of Kerala, Kasaragod, 671320, India; 2 Department of Genomic Science, Central University of Kerala, Kasaragod, 671320, India

**Keywords:** Carcinogenesis, Mitochondrial microRNAs (MitomiRs), Glycolytic switch, Electron Transport Chain (ETC)

## Abstract

Carcinogenesis is a multilevel process characterized by genetic and epigenetic alterations, thus contributing to uncontrolled proliferation that eventually leads to cancer. The process of carcinogenesis involves an intricate dis-orchestration in the expression of both, coding and non-coding sequences and is also dependent on the reprogramming of energy metabolism as both direct and indirect consequence of oncogenic mutations. Dysregulated mitochondrial energetics is an important hallmark of cancer, where cancer cells switch to the glycolytic pathway as an alternate source of energy to support the continuous energy supply needed for their indefinite growth. Even though functional mitochondria are indispensable for cancer cells, cancer cells exhibit different bioenergetics transitions based on the development status of cells undergoing carcinogenesis. Although the role of coding sequences in regulating energy metabolism shift is well studied, the role of non-coding sequences in modulating energy metabolism is still unclear. MicroRNAs (miRNAs), usually present in the nucleus and cytoplasm, have now been reported to localize in the mitochondria also known as, mitochondrial miRNAs (MitomiRs), which can originate either from the nuclear or mitochondrial genome. MitomiRs are reported to be associated with both oncogenic and tumor-suppressive functions. MitomiRs can target metabolic pathway-related protein-coding genes to alter cellular metabolism and promote carcinogenesis. Several mitomiRs like miR-1, miR-133, miR-128, and miR-21 have been reported to be involved in normal physiology, survival, and pathology. Since energy metabolism is one of the most important hallmarks of carcinogenesis and its underlying mechanism involves dysregulation of mitochondrial metabolism, we have tried to collate the importance of mitomiRs in the process of cancer energy metabolism and carcinogenesis.

## Introduction

Cancer is a disease of uncontrolled proliferation by transformed cells subject to evolution by natural selection. This recently updated definition of cancer by J.S. Brown et al. is consistent with the dynamic nature of cancer with the idea of natural selection [1]. It is a genetic disorder that arises through sporadic mutations in the genes of somatic cells or germline cells that lead to an altered pattern of gene expression which eventually disrupts normal cellular functions [2]. The term used broadly as ‘cancer genes’ refers to those genes mainly encoding the cell surface receptors, kinases, phosphatases, tumor suppressor genes, and transcription factors, where their altered expression fuels the process of oncogenesis via enabling various hallmarks of cancer [3].

Apart from the coding genes, non-coding genes play a crucial role in the initiation and progression of various cancers, where their transcription is contributed by approximately 75% of the total genome [4]. These untranslated transcripts, known as non-coding RNAs (ncRNA) are the key regulators of several physiological and pathological events, where they mediate chromatin remodeling, transcription, post-transcriptional modifications, and signal transduction. Among the diverse classes of non-coding RNAs, long non-coding RNA (lnRNA), microRNA(miRNA), circular RNA (circRNA), and PIWI-interacting RNA (piRNA) are reported to be involved in the pathological context of cancer [5].

miRNAs have been the subject of a multitude of research since their discovery in 1993 due to the tremendous versatility in their function and their ubiquitous role in gene regulation. miRNAs are generally 22 nucleotides in length which regulates the expression of mRNAs through post-transcriptional gene regulation. Multiple mRNAs can be targeted by a single miRNA, and on the other hand, different miRNAs can simultaneously target one mRNA [6]. Any miRNA that targets a protein involved in mitochondrial metabolism is referred to as mitochondrial microRNA (mitomiR) [7]. To regulate their targets in the mitochondria, mitomiRs exist in two distinct sets of miRNAs: the first set is encoded by nuclear genomes, which are transcribed in the nucleus, mature in the cytoplasm, and translocated into the mitochondria for regulating the mitochondrial gene. They are referred to as nuclear MitomiRs (Nuc-mitomiRs). The other one, known as mitochondrial mitomiRs (mt-MitomiR), is transcribed from the mitochondrial genome and controls the genes involved in the mitochondrial machinery.

Metabolic reprogramming by cancer cells, alternatively known as the Warburg effect, is one of the highlighted phenomena reported in tumorigenesis, where the cells shift their mitochondrial energetics from oxidative phosphorylation to glycolysis, even in the presence of oxygen, to meet the high energy demands and metabolic intermediates for rapid cell proliferation [8]. MitomiRs play a significant role in mitochondrial biogenesis, dynamics (fusion and fission), and the maintenance of mitochondrial DNA integrity where these processes are vital for mitochondrial health and function. Dysregulated mitomiRs expression can lead to altered energy metabolism, increased production of reactive oxygen species (ROS), and apoptosis resistance features commonly observed in cancer cells [9]. Understanding the regulatory networks of mitomiRs and looking into their specific targets may help us understand how they affect the development of cancer. Moreover, the key understanding of the role of mitomiRs may be used as therapeutic targets or as prospective biomarkers for the diagnosis and prognosis of cancer. This review delves into the connections between mitomiRs and carcinogenesis, highlighting the intricate regulatory mechanisms exerted by these small RNA molecules on mitochondrial physiology and their potential contributions to cancer development and progression.

## Cancer

In a broader sense, cancer refers to a collection of diseases that are of 277 distinct types [10]. It is one of the most fatal diseases around the globe, after cardiovascular disorder and it is predicted that there will be 611,720 cancer deaths and 2,001,140 new cancer cases in the US in 2024 [11,12]. Breast cancer, Colon cancer, and Hepatic cancer rank high, among the list of cancer cases reported worldwide [13]. Though cancer research was started several decades ago, no effective therapeutic strategy to control the growth of cancer has been discovered yet, for a majority of cancers, making it fall into a group of diseases, that claim maximum lives worldwide every year [14]. The underlying reason behind the lack of understanding of its mechanism of initiation and finding its specific cure is, that cancer exhibits variability at the tissue level, which poses significant challenges for both specific diagnosis and therapeutics [15].

Cancer is a disease of unregulated cell growth, where cancer cells acquire the ability to bypass all the anti-proliferative signals and divide in an uncontrolled manner [1], by accumulating multiple mutations in their genome [16]. The transformation of a normal cell to a cancerous one long and sequential process caused by a sequence of mutations in the genes that are responsible for cellular growth and differentiation [17]. Mutations are genetic alterations in the nucleotide sequence of the genes, caused by unrepaired DNA damage, by a variety of substances called mutagens, including chemicals, environmental factors, etc. [18].

### Types of cancer

**Figure 1:**
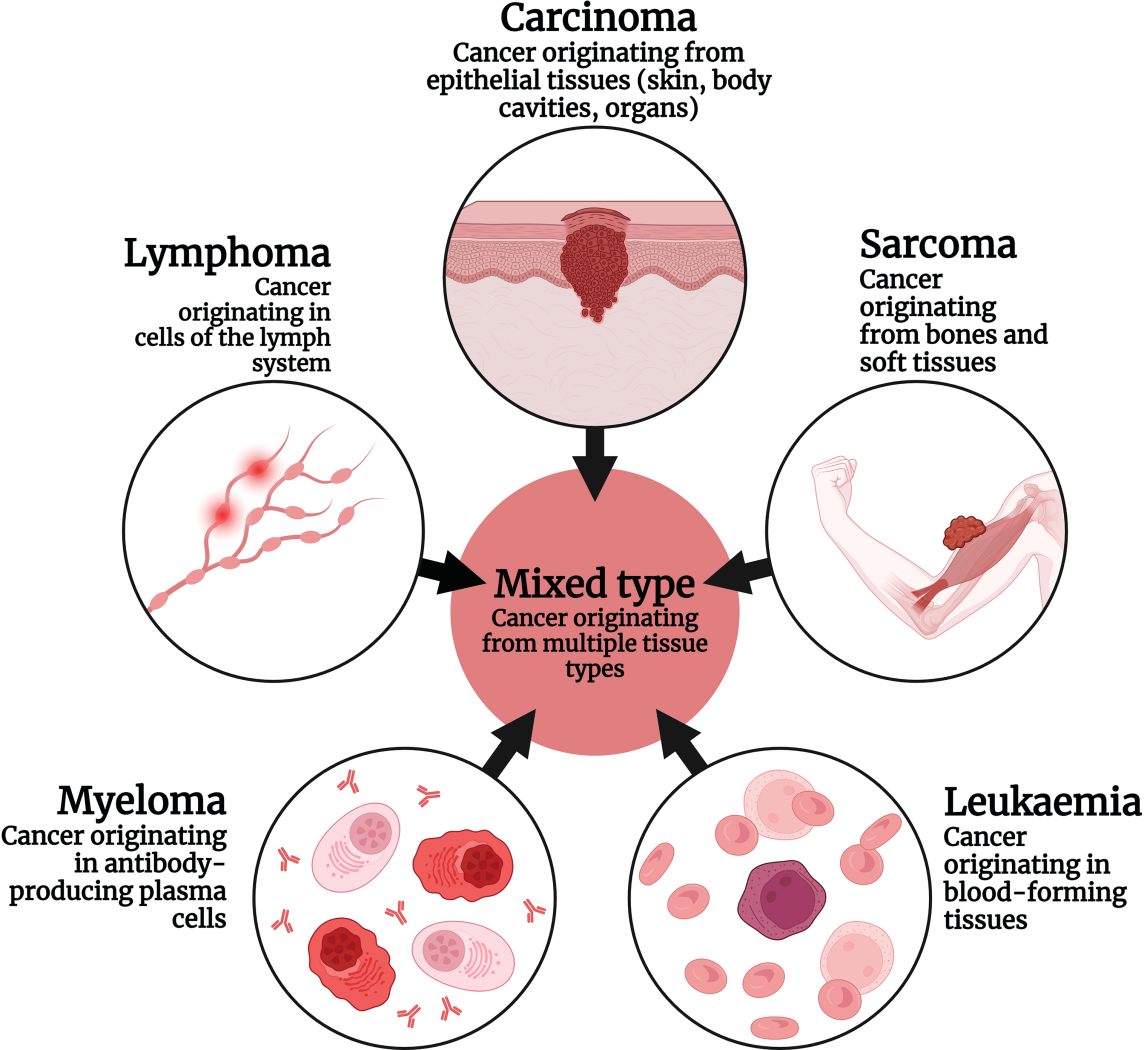
Types of cancer based on cellular origin. Created with Biorender.com.

Cancer has been primarily divided based on its cellular origin (Fig. 1). A few important subtypes are Carcinoma, Sarcoma, Lymphoma, leukemia, etc. Carcinomas are the most common type of cancer originating from epithelial cells, which coats the lining of the important organs including skin, colon, prostate, lung, and breast [19].

Carcinomas are further divided into two categories, adenocarcinoma (arising from glandular cells) and squamous cell carcinoma (originating from squamous cells, lining the organs). An example include hepatocellular carcinoma (HCC), breast cancer, lung cancer, etc. [20]. Sarcoma is are less common cancer type when compared to carcinoma. It originates from the connective tissues like bone, muscle, cartilage, etc. Osteosarcoma is the main type of sarcoma originating from bone cells (Primary), as well as metastasis from other cancers (Secondary) [21].

Leukemias are cancers originating from the blood-forming tissues, whereas lymphomas are malignancies developing from the lymphatic system, i.e., lymph node, spleen, etc. Apart from these types, tumors of the central nervous system (CNS), originating from brain cells, constitute a considerable portion of the total number of cancer cases, diagnosed every year [22]. Though a complete cure for a majority of cancers is not available yet, with the advancement in science and technology in the past few decades, the diagnosis and management of cancer have been made possible, providing a better chance of survival [23].

### Carcinogenesis

Boveri in 1914, hypothesized that cancer is cellular, arising from a single cell that has a chromosomal defect, which spreads to other cells during cell division, causing them to proliferate rapidly [24]. The onset of cancer is influenced by a wide range of internal and external elements, where genetic abnormalities, radiation exposure, starvation, infectious agents, and tobacco use, are some examples of external causes and the internal factors include hormone imbalances, genetic illnesses, and gene mutations [25]. The process of initiation and progression of cancer is referred to as carcinogenesis. It is a sequential process, starting with a series of premalignant stages (initiation), leading to invasive cancer (progression) [17].

The agents possessing the ability to induce carcinogenesis are called carcinogens, which could be of physical, chemical, or biological nature and they basically, are capable of damaging the DNA, resulting in mutations leading to carcinogenesis [26]. In 1775, Percival Pott made the first known observation linking soot exposure from chimney sweeps to scrotal cancer, leading to the first known study identifying the chemical exposure-related cause of cancer [27]. Subsequently, in 1874, Volksmann noted the elevated risk of skin cancer among tar workers, and Rehn in 1895 reported bladder cancer among workers exposed to aromatic amines. Since then, various carcinogens have been reported which include ethidium bromide (EtBr), tobacco, asbestos, aflatoxin, UV rays, IR rays, hepatitis virus, etc.

### Hallmarks of cancer

**Figure 2:**
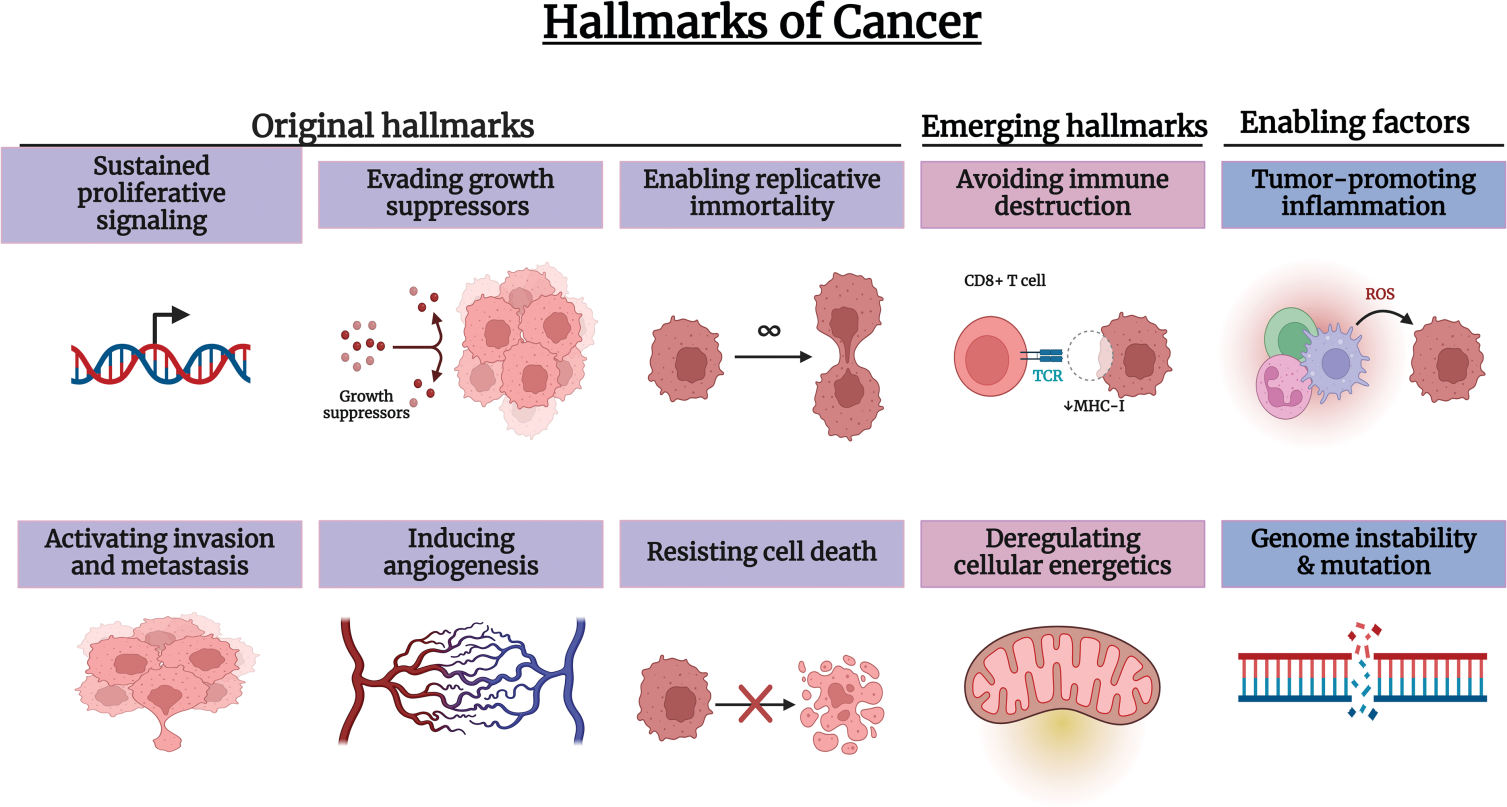
Hallmarks of cancer. Created with Biorender.com.

The hallmarks of cancer are the functional abilities that normal cells acquire as they transform into malignant cells (Fig. 2). The most important hallmarks observed in a variety of cancers include the following.

#### Sustained proliferation

The ability of normal cells to regulate their proliferation and maintain tissue homeostasis is dependent on growth signaling from a tightly controlled cell cycle, which is compromised in cancerous cells [28]. It is now understood that the growth and proliferative signaling pathways of cancer cells carry one or more driving changes inside their compartments (growth ligands, their receptors, cytosolic signaling molecules), which gives them an advantage over other cells in terms of survival. Deregulation of the cell cycle and disruption of checkpoints are essential for the growth of cancer cells [29]. The retinoblastoma (RB) protein is one important regulator that is frequently inactivated in a variety of cancers. Apart from the regulation of proliferation, the RB family is also involved in the maintenance of genomic stability, regulation of apoptosis, cellular metabolism, etc. [30].

#### Genetic instability

Normally, under the conditions of severe DNA damage, the cell employs multiple levels of DNA repair mechanisms amended for specific types of DNA lesions and these DNA repair pathways get defective in a variety of cancers, where cancer cells induce multiple mutations in the DNA repair genes [28,31].

#### Evading apoptosis

The usual balance between cell death and proliferation that ensures healthy tissue homeostasis is disrupted in cancer. Cancer cells bypass apoptotic signals either by mutational repression of p53 tumor protein (p53), upregulation of anti-apoptotic signals, or by loss of pro-apoptotic proteins [28,32].

#### Replicative immortality

Senescence is induced upon the crucial shortening of telomeres after the exhaustion of replication potential in normal cells. Short telomeric length is an almost ubiquitous characteristic of all pre-malignant tumors, but at their advanced stages, the tumors bypass this senescence by synthesizing telomere for further continued replication, with the help of telomerase enzyme, which has been found 85%–90% upregulated in a variety of cancers [28,33].

#### Angiogenesis

A tumor cannot spread or develop larger than 2–3 mm, without new blood vessels. The process of formation of new blood vessels is called angiogenesis [34]. Following hypoxia condition, the cancer cells induce angiogenesis to adequately supply the nutrients and oxygen to the developing tumor, to support its proper growth and proliferation. To achieve this, they suppress anti-angiogenic factors that prevent blood vessel formation and induce the production of pro-angiogenic factors that escalate blood vessel formation [28,35].

#### Invasion and metastasis

The most widely recognized characteristic feature of cancer cells is their capacity to infiltrate adjacent tissues. The ability of cancer cells to metastasize is a result of numerous alterations that they undergo. The process consists of several steps, the first of which is the local invasion of the cells into the surrounding tissues. The epithelial cells exhibit immortality and exhibit strong adhesion to both the surrounding matrix and one another [36]. Overcoming such obstacles is associated with the reversible biochemical changes known as the epithelial-mesenchymal transition (EMT), which enable polarized epithelial cells to take on the characteristics of a mesenchymal cell [37]. After that, they evade and enter blood vessels called circulating tumor cells, endure the hostile environment of the circulatory system, leave it, and begin to divide in the new tissue [38].

#### Immune invasion

The immune system’s ability to eradicate and modify malignant tumors is known as cancer immune editing and it consists of three stages: elimination, equilibrium, and escape. At an advanced stage, the tumor continues to grow with a properly functional immune system, despite tight immune surveillance [39]. Though cancer cells go through constant immune selection pressure, some cancer cells emerge and ‘escape’ immune surveillance and grow in an uncontrolled manner resulting in tumors. By secreting immunosuppressive substances or enlisting the help of immunosuppressive inflammatory cells, cancer cells can neutralize the immune system’s cytotoxic components [40].

#### Altered mitochondrial metabolism

The dysregulated mitochondrial mechanism is an important hallmark of cancer, where the mitochondrial metabolism is altered and the glycolytic pathway is activated as an alternate source of energy, in order to support the exponential growth of the tumor cells [28]. To achieve this, along with preventing mitochondria from completing normal aerobic respiration (oxidation of pyruvate, the citric acid cycle, and the electron transport chain), cancer cells upregulate glycolysis and lactic acid fermentation in the cytosol [41].

The mitochondrial metabolism could be altered either by the mutations in the genes involved in energy production or by the suppression of the expression of genes involved in the normal functioning of mitochondria. Tumor suppressors or oncoproteins rewire mitochondrial activity to promote the growth and maintenance of tumors [42]. Within mitochondria, certain oncoproteins or tumor suppressors such as Iso-citrate dehydrogenase NADP+1 (IDH1), Succinate dehydrogenase (SDH) & Fumarate hydratase (FHs), produce oncogenic metabolites, i.e., succinate and fumarate and 2-hydroxyglutarate, that are necessary for tumor initiation and several others affect the mitochondrial metabolic machinery directly or indirectly [43].

In cancer, mitochondria play a crucial role in tumor growth by enhancing energy metabolism, limiting mitochondrial-induced apoptosis, and increasing ROS-mediated DNA damage leading to mutations [44].

## Genes Involved in Carcinogenesis

Unrepaired DNA damage leads to multiple mutations in the genes associated with cellular proliferation, resulting in an inappropriate over-expression of oncogenes, by down-regulating or disabling tumor suppressor genes [45]. The coding sequence as well as the non-coding DNA sequence play an important role in the process of carcinogenesis. On one hand, the coding sequences code for the genes directly involved in carcinogenesis, i.e., oncogene and tumor suppressor genes (TSG), and on the other hand, non-coding sequences, including miRNAs play a crucial role in the regulation of the expression pattern of oncogenes and TSGs [46].

### Coding sequence

The nucleotide sequences that carry genetic information for encoding a protein are called coding sequences or genes. To transform a normal cell into a cancer cell, the genes that regulate cell growth and differentiation must be altered. The affected genes are divided into two broad categories i.e., oncogenes and TSG. Oncogenes are a class of genes that promote cellular growth and differentiation, whereas TSG is the genes that inhibit cell division and survival, e.g., P53, RB, etc. [45,47]. A variety of TSG & oncogenes have been reported to be involved in a wide range of cancers like lung, breast, Hepatocellular carcinoma (HCC), etc., e.g., K-Ras, Catenin beta 1 (CTNNB 1), B-cell lymphoma/lymphoma 2 (BCL2), Phosphatase and tensin homolog (PTEN), p53, RB, etc. [48–50]

### Non-coding sequence

The RNAs, that do not get translated into protein, are called noncoding RNAs, which include tRNA, rRNA, lnRNA, and small noncoding RNAs such as siRNAs and miRNAs. Various reports have revealed the direct involvement of non-coding sequences in the process of carcinogenesis [51]. The non-coding RNAs like nRNA, PiRNA &miRNAs possess regulatory properties, where they are capable of regulating the expression levels of their target genes [46,52].

#### ln RNA

lnRNAs regulate the expression of a variety of genes involved in cancer and may possess oncogenic or tumor suppressive role [53]. Oncogenic lnRNA includes Transcription factor-7 (TCF7) which has been found at higher levels in liver cancer and was important for self-renewal [54]. Also, lnRNA epigenetically-induced lncRNA1 (EPIC1) has been found at elevated levels in liver, breast, lung & colon cancer and has been reported to promote carcinogenesis [55].

Some lnRNAs also have been reported to exhibit tumor suppressor function, for example, lnRNA, Disrupted In Renal Carcinoma 3 (DIRC3), has been reported to act as a melanoma tumor suppressor, by acting locally to block SRY-box transcription factor 10(SOX10) chromatin binding at melanoma regulatory elements and activate Insulin-like growth factor binding protein 5 (IGFBP5) expression, a tumor suppressor, thereby inhibits the proliferation of melanoma cells [56].

#### PiRNA

Approximately 24–31 nucleotides in length, PIWI-interacting RNAs (piRNAs) are a less-studied type of short non-coding RNAs. They exhibit expression in both germline and somatic cells, they interact with PIWI proteins to perform regulatory functions [57]. Also called piwiRNAs, they have been reported to play a crucial role in the suppression of transposable elements, de-adenylation, and decay. Several malignant tumors and important cancer hallmarks such as enhanced stemness, invasion, metastasis, and decreased apoptosis, have been linked to aberrant expressions of piRNAs and PIWI proteins. Additionally, several pathways of piRNA-mediated target dysregulation linked to the development, spread, or beginning of cancer have been shown by various studies [58]. piRNA-823 has been reported to be involved in cell migration and proliferation and its inhibition suppressed cell proliferation in colon cancer [59].

#### MiRNAs

miRNAs are small transcripts of approximately 22 nucleotides, involved in the regulation of gene expression at post-transcriptional and translational levels by directly binding to the 3’-UTR or the binding site on the target mRNAs. miRNAs are regarded as negative regulators of their target genes and they play a crucial role in regulating numerous biological processes such as development, differentiation, proliferation, DNA repair, and apoptosis in humans since they are capable of influencing a majority of genes [60].

miRNAs have the ability to target multiple mRNAs, and therefore can concurrently control several target genes within the same pathway or even throughout distinct pathways and the physiological state of a cell is frequently determined by the tissue-specific patterns of miRNA expression [61].

##### Biogenesis of miRNAs

**Figure 3:**
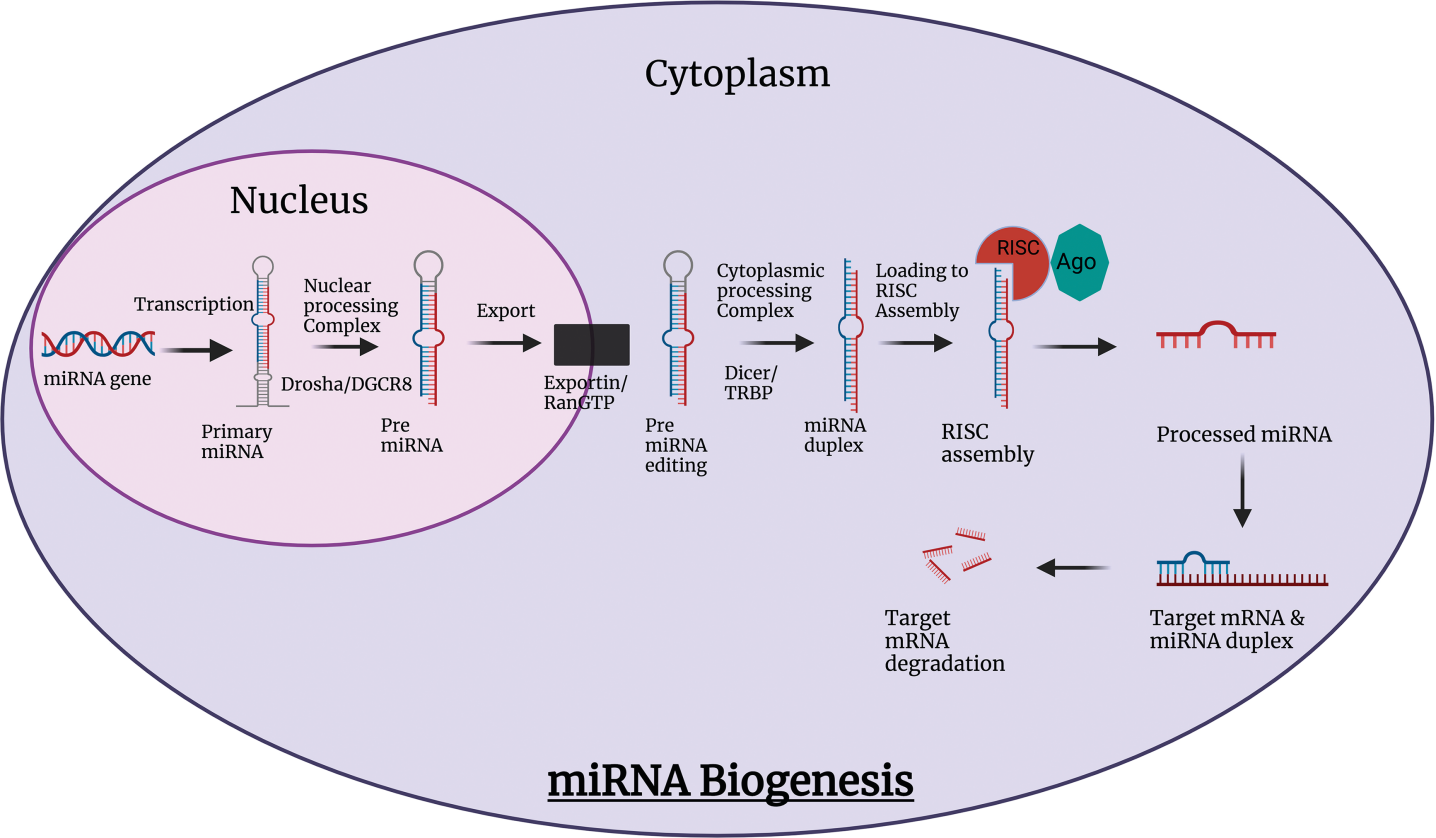
miRNA biogenesis. Created with Biorender.com.

The synthesis of miRNAs in cells is a multiphase, coordinated process that begins in the nucleus, moves through the cytoplasm, and ends with the creation of the biologically active form of miRNA (Fig. 3) [62].

The biogenesis of miRNA starts with the synthesis of a long transcript known as primary miRNA (pri-miRNA) which is transformed into intermediate precursor miRNA (pre-miRNA) hairpin by sequential biochemical processes, where at first step, pri-miRNAs are transcribed by RNA polymerase II [63]. This pri-miRNA is cropped into a 70 nucleotide hairpin structured, precursor miRNA (pre-miRNA), catalyzed by a multi-protein complex called a microprocessor. The core components of the microprocessor complex are Drosha, an RNase III enzyme, together with its interacting partnerDiGeorge syndrome critical region 8(DGCR8), a double-stranded RNA-binding domain (dsRBD) protein [64].

In order to release the pre-miRNA, Drosha cuts the stem around 11 nucleotides away from the stem-ssRNA junction using the DGCR8 protein, which detects the stem and the flanking single-stranded RNA. The pre-miRNA is then transported to the cytoplasm through the interaction with exportin-5 and Ran-GTP [65].

The pre-miRNA is cleaved by Dicer, to produce about 22 nucleotide double-strand (ds) RNA containing a mature miRNA and a passenger miRNA strand. Mature miRNA, together with dicer, initiates the assembly of the RNA- induced Silencing Complex (RISC), a ribonucleoprotein complex [66]. Once incorporated into the RISC, the miRNA guides the complex to its targets by base-pairing interactions. In cases of perfect or near-perfect complementarities to the miRNA, target mRNAs can be cleaved (sliced) and degraded, otherwise, their translation is repressed [67].

##### Role of miRNAs in physiology

The importance of faithful miRNA expression has been involved in numerous cellular events during animal development where it is pivotal for the regulation of many key processes such as cell fate determination, proliferation, and cell death [68]. For example, miR-196 is involved in hind limb development [69]. Other examples include the brain-specific miR-134, which is necessary for synaptic development and plasticity [70]. Skin differentiation is promoted by miR-203, which represses p63 in epithelial tissues, while precise levels of miR-1 are critical in cardiac development [71]. Normal immune function is dependent on miR-155 and B-cell differentiation is controlled by miR-150 mediated repression of the transcription factor c-Myb [72]. The pancreatic islet cell-specific miR-375 regulates insulin secretion by inhibiting myotrophin of the exocytosis 7 pathway [73].

Various studies have shown that the miRNAs also play a key role in the growth, survival, and functioning of neurons, thereby regulating nervous system functioning. miR-133b regulates the maturation and function of the dopaminergic neurons in the midbrain, involved in locomotion control [74].

Both, loss or dysregulation of specific miRNAs and genetic ablation of the miRNA machinery seriously impair immune development and increase the risk of immunological diseases such as cancer and autoimmune [75]. It has been found that miR-223 is involved in the inflammatory response and is considered to be the fine-tuner of granulocyte production [76] and miR-146a was found to be a negative regulator of the interferon pathway [77].

miRNAs have also been found to be involved in the regulation of reproductive functions such as embryo development, oocyte maturation, and luteum development [78]. Various groups have reported the differential expression of several microRNAs at different stages of testis and oocyte development. The list of miRNAs regulated physiological function is many and has been beautifully summarized in “Role of miRNAs in physiological and pathological processes” by Tomankova et al. [79].

##### Role of miRNAs in pathology

Through a number of different mechanisms, the dysregulation of miRNAs has been connected to several disease conditions, i.e., cardiovascular disease, diabetes, angiogenesis, cancer, etc. [80]. miR-21 has been found to regulate the ERK-Map kinase signaling pathway in cardiac fibroblasts, affecting its structure and function [81]. Also, miR-145 has been reported to regulate the fate of smooth muscle, and miR-145 along with miR-143 controls the proliferative/quiescent phenotypes of smooth muscle cells [82]. Studies have shown that miR-1, miR-133, and miR-208 are linked with heart development and regulation of myocyte differentiation and dysregulated expression of miR-1 and miR-133 was reported in heart failure [83]. miRNAs have also been reported to be involved in diabetes as they regulate the expression of gene cascade involved in pancreas development, e.g., miR-7 and miR-375 have been found at higher levels in the pancreas and are responsible for pancreas development and insulin secretion [84]. Various studies revealed that over-expression of miR-210 in endothelial cells resulted in increased angiogenesis and miR-221 and miR-222 exhibited anti-angiogenic effects [85]. Along with this, several studies have established the relation of various miRNAs with numerous disease conditions, i.e., miR-29 in neurodevelopmental disease [86], miR-146 in rheumatoid arthritis [87], etc. The list of diseases that are either caused or programmed due to dysregulated miRNA-mediated regulation is long and has been summarised in “Role of miRNAs in human disease” by Ardekani et al. [88].

## Role of miRNAs in Cancer

Apart from regulating the normal functioning of the cells, tissue/organ development, and implications in various disease conditions, miRNAs also play a very crucial role in cancer initiation and progression. The first report linking miRNAs to cancer was on the differential expression of miR-15 and miR-16 in chronic lymphocytic leukemia (CLL), which established the relations of these two miRNAs with the pathogenesis of CLL [89]. The miRNAs involved in carcinogenesis fall into two broad categories, i.e., oncomiR, and tumor suppressor miR [90].

OncomiRs target the tumor suppressor genes (TSG) and promote carcinogenesis. For example, the levels of miR-126 were found high in colon cancer [91], where it was reported to suppress the expression of P53 [92]. Similarly, the oncogenic role of miR-155 has been found in liver cancer [93]. Tumor suppressor miR inhibits cellular proliferation by targeting the genes involved in cell growth and differentiation, e.g., miR-let-7 family has been reported as a tumor suppressor and found to be downregulated in various cancers like HCC and pancreatic cancer [94,95].

The involvement and the specific role of the miRNAs have been indicated in various types of cancer including breast, colon, lung, and gastric cancer etc. For example, the miR-let-7 family has been reported to inhibit the expression of RAS, and downregulation of miR-let-7 in lung cancer leads to overexpression of RAS and contributes to oncogenesis [96]. miR-9 has been found to be upregulated in breast cancer cells and promote cell motility and invasiveness by directly targeting E-cadherin, cadherin 1 (CDH1) [97]. Similarly, miR-155 has been reported to suppress apoptosis in T-cell leukemia and breast cancer [98].

In gastrointestinal cancers, various miRNAs have been found regulated suggesting they may have a role as tumor suppressors, e.g., miR-15b and miR-16 which are downregulated in gastric cancers, have been associated with multi-drug resistance by regulating apoptosis via targeting the expression of BCL2 [99]. Also, elevated levels of several miRNAs have been reported to be directly involved in gastric cancer, e.g., miR-135a and miR-135b are found at high levels in colorectal cancer, where they target the APC gene and induce the Wnt signaling pathway [100]. This is just a very brief account of the role of miRNAs in cancer. The extensive information about this topic has been summarized in Peng et al. [101].

## Role of miRNAs in Cancer Stem Cells (CSCs) Carcinogenesis

Stem cells are a pool of precursor cells that exist in an undifferentiated form and have the unique ability to self-renew and undergo asymmetrical division, promoting normal cell proliferation. The typical cellular features of stem cells are governed by various factors including, differential gene expression under epigenetic, transcriptional, translational, and post-translational regulation, as well as signals from nearby cells [102]. Any error in these regulatory circuits, leads to the accumulation of aberrant epigenetic modifications, inducing the signaling pathways that facilitate carcinogenesis, free the cells from the confines of their niche, and turn them into cancer stem cells (CSCs), which structurally & functionally differ from other cells in the tumor mass and possess the ability to undergo self-renewing mitosis, in which one of the daughter cells becomes a progenitor cell and the other a stem cell. Along with this, the capacity of CSCs to initiate tumor growth, multiply, infiltrate, migrate, and resist therapeutic actions is another attribute, that sets them apart [103].

Cancer stem cells exhibit a dynamic expression profile of miRNAs, indicating their importance in regulating the processes that lead to increased tumor initiation and metastatic potential, such as migration, invasion, exit & differentiation from the cell cycle, prosurvival& anti-stress mechanisms (such as resistance to anoikis) and epithelial-mesenchymal transitions (EMT) [104]. Several studies have demonstrated the role of various CSC-related miRNAs in cancer, which are summarized in Table 1.

**Table 1:** CSC-associated miRNAs in various cancers

miR	OncomiR/TS miR	Cancer type	Reference
miR-17-92 polycistron	Onco miR	Upregulated in lung, breast, stomach, prostate, colon, and pancreatic cancers	[105,106]
miR-181	OncomiR	Hepatocellular carcinoma	[107]
miR-29	TS miR	Cholangiocarcinoma	[108]
miR-126	Onco miR	Gastric carcinoma	[109]
Let-7		Colon adenocarcinoma	[110]
Let-7	TSmiR	Hepatocellular carcinoma	[107]
miR-125b	TS miR	Glioma	[111]
miR-21, miR-205	Onco miR	Head and neck cancer	[112]
miR200c	TS miR	Head and neck squamous cell carcinoma	[113]
miR-21	Onco miR	Breast cancer	[114]
miR-495	Onco miR	Breast cancer	[115]
miR-15 miR-16 cluster	TS miR	Chronic lymphocytic leukemia	[106]
miR-372, miR-373	Onco miR	Testicular germ cells	[116]

## Localization of miRNAs

miRNA expression is versatile in nature and varies from tissue to tissue and organelle to organelle. Apart from their differential expression pattern, miRNAs exhibit localization capabilities, i.e., they get targeted from cytoplasm to the mitochondria and endoplasmic reticulam [117,118]. The existence of miRNAs in the mitochondria and their critical roles in the control of mitochondrial activities and local mitochondrial protein synthesis has been revealed by a number of studies. Initially, in 2009, 5 mitochondrial enriched miRNAs (miR-130a, miR-130b, miR-494, miR-140, miR-320) were identified in rat liver cells, followed by the first report regarding the localization of miRNAs to the mitochondria of human primary skeletal muscle cells in 2011 [119]. Later, in the same year, a study on mitochondrial miRNA enrichment in Hela cells revealed that 57 miRNAs were significantly deregulated in the mitochondria when compared with the cytosol and three nuclear-coded miRNAs (miR-494, miR-1275, and miR-1974) were found significantly enriched in the mitochondria [120]. Das et al. demonstrated that miR-181c has a specific motif at the 3’-terminal end for mitochondrial translocation [121]. A summary of the mitochondrial localized miRNAs with their functions is presented in Table 2.

**Table 2:** Localized/Enriched mitochondrial miRNAs and their functions

Sr. No.	MiRNAs	Functions	References
1	miR-494	Affects ATP synthesis and leads to mitochondrial dysfunction	[124]
2.	miR-132	Inhibits complex 1 of mitochondria and reduces aerobic respiration	[125]
3.	miR-34a	Synaptic plasticity dysfunction	[126]
4.	miR-365	Accumulation of ECM components and secretion of inflammatory cytokines	[127]
5.	miR-338	Reduces mitochondrial oxygen consumption	[128]
6.	miR-484	Inhibits mitochondrial fission	[129]
7.	miR-218	Inhibits mitophagy and mitochondrial clearance	[129]
8.	miR-212	Enhance neurotransmission and be involved in synaptic plasticity	[125]
9.	miR-146a	Induces inflammatory response and contributes to cell aging	[130]
10.	miR-130a	Induced neurotoxicity and cell apoptosis	[124]
11.	miR-130b	Inhibits cell proliferation and induces cell apoptosis	[124]
12.	miR-140	Inhibits mitochondria dysfunction and enhances autophagy	[124]

Most mitomiRs are transcribed in the nucleus, but some, such as miR-1974, miR-1977, and miR-1978, are encoded by mtDNA [122]. The discovery of pre-miRNA and the protein AGO2 within the mitochondrion raises the possibility that some mitomiRs’ synthesis may occur there and that AGO2 may also impede the transit of these molecules into the interior of the mitochondria. However, there is no evidence regarding the presence of the miRNA biogenesis machinery inside the mitochondria [123].

## Mitochondrial Genome

**Figure 4:**
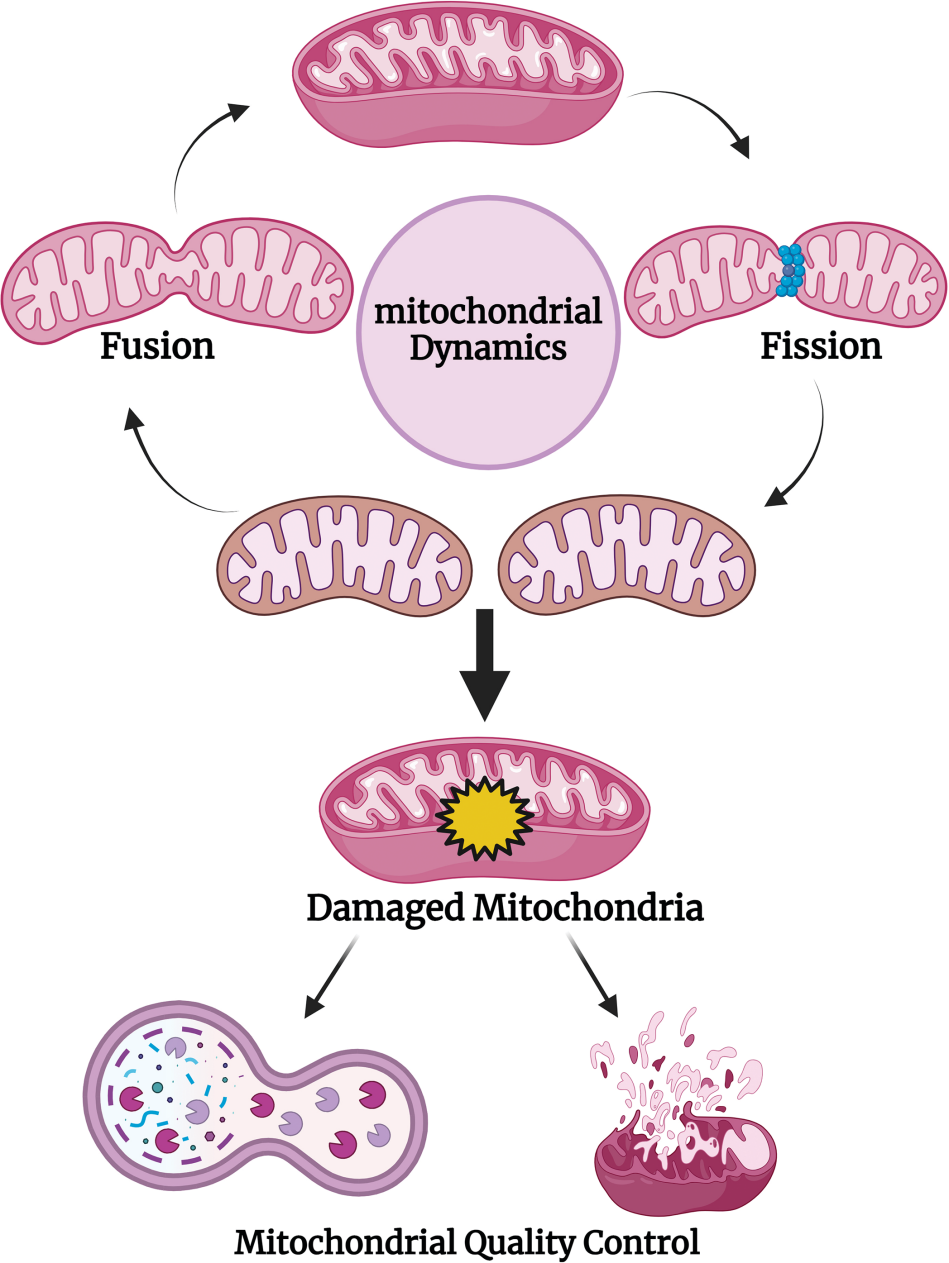
Mitochondrial dynamics and its quality control. Created with Biorender.com.

Mitochondria possess their own genetic material called mtDNA, which consists of 13 protein-coding genes. These genes are directly involved in ATP production by, etc, comprising oxidative phosphorylation (Ox-Phos), crucial for multicellular organisms. Along with 13 protein-coding mRNAs, mtDNA consists of 2 rRNA, 22tRNA, and several non-coding RNAs, which are called mitochondrial microRNAs [131]. Mitochondrial homeostasis is maintained by mitochondrial quality control, which assures the removal of damaged mitochondria (Fig. 4).

Various studies suggest that nuclear-coded miRNAs get localized to the mitochondria, express within mitochondria, and exert their function on target mitochondrial genes [129]

### MitomiRs

While most miRNAs are primarily found in the cytoplasm, a subset of them has been discovered within mitochondria, referred to as mitochondrial miRNAs or mitomiRs. These mitomiRs are thought to control gene expression within the mitochondria or have an indirect effect on mitochondrial function [117].

MitomiRs could be either of mitochondrial origin or of nuclear origin that are localized in mitochondria. The fact that miRNAs are found in mitochondria indicates that they may have a role in regulating the expression of genes, metabolism, and other functions within the mitochondria [132]. They could affect mitochondrial function by interacting with mitochondrial proteins or by controlling the translation of mitochondrial mRNA. Certain mitomiRs have been demonstrated to concurrently target nuclear-encoded mitochondria-associated mRNAs and various mitochondria mRNAs, rRNAs, and tRNAs. A number of human disorders associated with mitochondrial malfunction have been linked to aberrant expression of mitomiRs, which are crucial modulators of mitochondrion tasks [133].

### Role of mitomiRs

miRNAs coded by nuclear DNA as well as by mitochondrial DNA have targets on mitochondrial protein-coding genes. miRNAs, which translocate from the nucleus/cytoplasm to mitochondria are called MitomiRs. These mitomiRs either target membrane proteins involved in ATP transportation outside the mitochondria or internal proteins, which are directly involved in ATP synthesis, so they are considered key players in various diseases associated with mitochondrial malfunctioning including cancer. Any physiopathology requires some biological process that gets modulated by mitomiRs like miR-1, miR-133, miR-128, miR-21, etc. [134].

#### Survivability

miRNAs have a strong impact on the survivability of an organism, as they regulate the expression pattern of almost all the genes involved in the normal functioning of the cellular machinery of an organism. Several miRNAs are reported to have targets on important mitochondrial genes. Mitochondrial malfunctioning by these miRNAs results in various disease conditions like muscular dystrophy, diabetes, cancer, etc. It’s well known that mitochondrial damage leading to its malfunctioning is primarily caused by mitochondrial miRNAs because they break down respiratory chain complexes and produce more ROS [129]. Several studies revealed that mitomiRs (miR-34a, miR-146a) play a vital role in the normal functioning of the central nervous system, where deregulation of mitomiRs results in immune response in the brain and the mitochondrial damage leads to the inflammation of neuronal synapses [135]. Similarly, miR-378 has been reported to be involved in the regulation of mitochondrial metabolism and homeostasis of the organisms by targeting carnitine O-acetyltransferase(CRAT), a crucial mitochondrial enzyme associated with fatty acid oxidative metabolism through Tricarboxylic acid (TCA cycle) [136].

#### Diseases

The mitochondrion is a crucial part of cells that are involved in metabolism, signaling cascades, bioenergetics, and cell viability. Dysfunctional mitochondria have been reported to be the underlying cause of the onset of various diseases. Mitochondrial diseases are primarily genetic disorders that can impact the neurological system, heart, kidneys, lungs, skeletal muscles, and more [137].

For example, a novel marker for the onset and progression of Alzheimer’s disease is mitochondrial dysfunction, according to reports [138,139], as it plays a crucial role in brain cells especially neurons, like providing synaptic energy (ATP), Ca^2+^ handling, apoptosis and ROS production [140]. Several examples of mitochondrial miRNAs associated with various neurological disorders are miR-494, miR-705, miR-155, miR-223, etc. [141]. Along with neurological disorders, mitomiRs play a crucial role in cardiovascular disease and examples include miR-181c, miR-21, miR-378, etc. [142]. Other diseases caused due to the dysfunction of mitochondria include kidney failure [143], respiratory failure, aging [144], GI tract disorder [145], visual loss [146], etc. A detailed list of important mitomiRs involved in various diseases has been briefly summarized in Table 3.

**Table 3:** List of mitomiRs in various diseases

Sr. No.	miRNAs	Diseases	Reference
1	miR-210	Diabetes	[147]
2	miR-137	Schizophrenia, Hemorrhagic stroke	[147]
3	miR-132, miR-196a	Huntington’s disease	[141]
4	miR-27a	Lung disease, Fatty liver disease	[147]
5	miR-30	Cardiac hypertrophy	[148]
6	miR-149, mi-494, miR-761	Various metabolic diseases	[134]
7	miR-34	Age-related cataract	[134]
8	miR-184, miR-338	Fabry Disease	[149]
9	miR-193b, miR-181a	Aging	[141]
10	miR-1	Myogenesis	[117]
11	miR-21	hypertension	[117]
12	miR-17	Polycystic kidney disease	[134]
13	miR-29a	Intracranial aneurysm	[134]
14	miR-181a	Liver fibrosis	[147]
15	miR-130b-3p	Gestational diabetes	[147]
16	miR-20a-5p	Kidney injury	[147]
17	miR-15a, mi-23a/23b, miR-330, miR-424	Alzheimer’s disease	[141]
18	miR-7, miR-21, miR-29a	Parkinson’s disease	[141]
19	miR-155a-5p	Asthenozoospermia	[134]
20	miR-7	Neurodegeneration	[134]
21	miR-181c	Heart failure	[117]
22	miR-378	Diabetic cardiomyopathy	[117]
23	miR-570	Platelet disease	[134]
24	miR-4331	Retinoblastoma	[134]
25	miR-378	Diabetic Heart	[148]
26	miR-210	Ischemic heart disease	[148]
27	miR-378	Obesity and metabolic syndrome	[134]

#### Metabolic glycolytic switch & carcinogenesis

One distinct biochemical feature of cancer cells is a switch in glucose metabolism from oxidative phosphorylation to aerobic glycolysis [150]. Numerous investigations have indicated that modifications in mitomiRs expression level are involved in human cancers, including the genesis, progression, and metabolic reprogramming of human cancers [151] (Table 4). Warburg originally hypothesized that glycolysis is an alternate pathway of energy production exhibited by most of the cancer cells at the advanced stages. He demonstrated that cancer cells go through a fermentation phase as opposed to aerobic energy metabolism and this metabolic reprogramming, dubbed the Warburg effect, accelerates the production of lactate and glycolysis [152]. Various studies showed that, in most cancers, the cancer cells modify their mitochondrial machinery and activate the glycolytic pathway to increase their metabolic rate to sustain rapid growth and proliferation [153,154] (Table 5).

**Table 4:** MitomiRs in various cancers

Micro RNAs	Type of cancer	Reference
miR-210	Lung cancer	[168]
miR-17-3p	Prostate cancer	[169]
miR-181c	Biliary tract cancer	[170]
miR-195	Breastcancer	[171]
miR-548b-3p	Hepatocellular carcinoma	[172]
miR-98	Bladder cancer	[173]
miR-483-5P	Tongue cancer	[174]
miR-210-5p	Colon cancer	[175]
miR-509-5p	Breast cancer	[176]
miR-31	Oral carcinoma	[177]
miR-26	Laryngeal cancer	[178]
miR-320	Cervical cancer	[179]

Note: *Adapted from [134] and modified.

**Table 5:** List of mitomiR involved in glycolytic switch and carcinogenesis

No.	miR	Mitochondrial target gene	Cancer	Biological impact	Reference
1.	miR-181a-5p	Cyto-B, Cox2	Liver cancer	High glucose consumption High cellular survivability under Hypoxic conditions	[180]
2.	miR-181c	Cox1	Colon cancer	Reduced mitochondrial Ox-Phos	[181]
3.	miR-2392	ND4,Cox1,Cyto-B	Tongue cancer	High glycolysis Reduced Ox-Phos	[182]
4.	miR-let-7a	ND4	Breast cancer	Increased glycolysis Carcinogenesis	[183]
5.	miR-1	ND4, ND1, Cox1, ATP6	Breast cancer, Melanoma	Enhance ATP production Carcinogenesis	[184]
6.	miR-5787	Cox3	Tongue cancer	High glycolysis Reduced Ox-Phos	[185]

Note: *Adapted from [7,186] and modified.

MitomiRs regulate tumor metabolism by regulating various pathways controlled by several receptors, growth factors, and tumorigenic proteins [155]. An important example of this is, the PI3K/Akt pathway and the hypoxia-induced factor (HIF-1α), whose significant contribution to the absorption of glucose and the glycolysis process in tumor cells has been established [156]. Several groups have shown the direct involvement of mitomiRs in the regulation of glycolysis, e.g., miR-155 was found to alter the energy consumption in breast cancer by upregulating hexokinase 2 (HK2), a glucose

**Figure 5:**
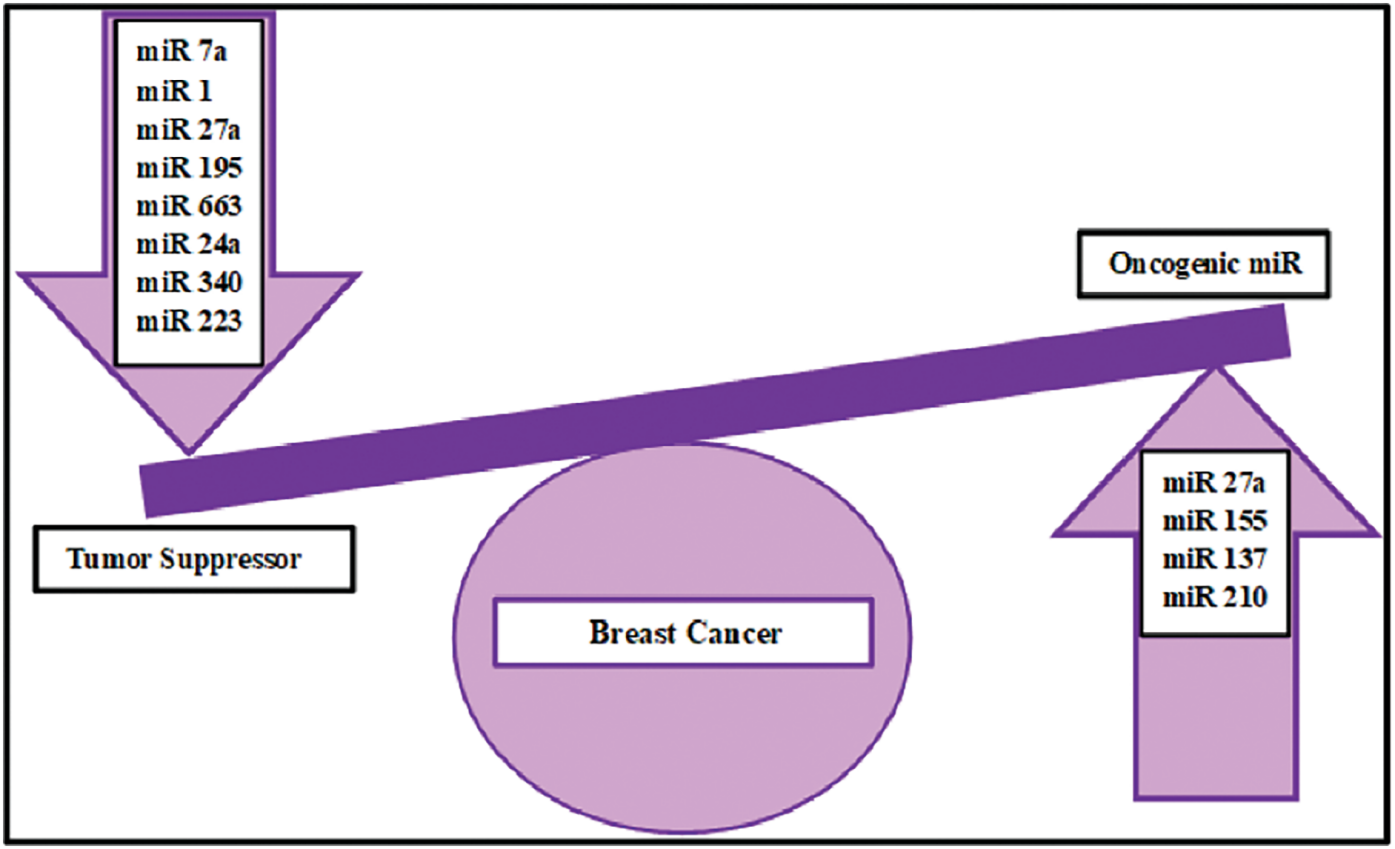
MitomiRs involved in breast cancer.

Phosphorylation enzyme involved in glycolysis [157]. Another miR, i.e., miR-210 targets the enzymes crucial for mitochondrial metabolism and thus contributes to the metabolic reprogramming in breast cancer [158] (Fig. 5)

Likewise, miR-124, miR-137 & miR-340 have been found to influence the growth of colorectal cancer by targeting pyruvate kinase isoenzyme (PKM), thereby regulating glucose metabolism [159]. Similarly, miR-326 acts as a potential regulator of glucose metabolism by regulating pyruvate kinase [160].

miR-126 has been found to suppress mesothelioma tumors by modulating mitochondrial energy metabolism [161]. Another mitomiR-210 has been implicated in the regulation of mitochondrial machinery under hypoxic conditions, where oxidative phosphorylation (OXPHOS) is replaced by glycolysis [162], and also, miR-210 has been connected to mitochondrial dysfunction in lung cancer. Apart from the regulation of mitochondrial machinery, mitomiRs are also involved in tumor initiation, progression, and apoptosis. For example, miR-200 has been reported to be involved in breast cancer progression [163] and the miR-17-92 cluster has been involved in the suppression of apoptosis [164] and metabolic reprogramming in various cancers like breast, liver, kidney, lung, etc. [165]. The ability of tumor cells to resist death is largely dependent on mitochondria, which are particularly involved in preventing apoptosis [166]. Various studies have demonstrated that miRNAs, i.e., miR-21, miR-155, miR-27a, are found to be involved in evading the apoptosis in breast cancer following mitochondrial pathways [167]. A summary of the mitomiRs involved in various types of cancer has been elucidated in Table 4.

A brief summary of the mitomiRs involved in glycolytic switch and carcinogenesis has been presented in Table 5.

## miRNA Therapeutics in Cancer

As discussed above, cancer is an aggressive disease that poses many challenges to treat because of a number of factors, including significant inter and intra-tumor heterogeneity, mutations in hundreds of different genes, the fact that it affects a variety of body organs and cells, including stromal, epithelial, and blood cells. Additionally, cancer is typically not a static disease but rather changes and progresses over time, accumulating new mutations [187].

There has been a notable advancement in cancer therapy in recent years due to the extraordinary breakthrough made in understanding the initiation and progression of cancer. The conventional treatment of cancer falls into three major categories namely chemotherapy, radiotherapy, and surgery [188]. In chemotherapy various drugs, inhibiting cell division and proliferation are used [189], whereas in radiotherapy cancer is treated by exposing the tumor to various radiations capable of destroying the cancer cells, which come in its proximity [190]. Surgery is mainly used for solid tumors and it is coupled with chemotherapy & radiotherapy, to reduce the size of the tumor before operating it [191]. In the advanced stages of a variety of cancers, combination therapy is given to the patients which consists of more than one type of therapy [192].

Various other novel therapies have cropped up that include; 1.) Immunotherapy is a cancer treatment technique that stimulates the immune system to target and destroy cancer cells to aid the body’s defenses against the disease [193]. 2.) Hormone therapy combats cancer, by altering the body’s hormone levels, especially those cancer types that are dependent on these hormones to proliferate and become more prevalent, i.e., Breast, prostate, and ovarian cancer [194]. 3.) Targeted therapy destroys cancer cells with drugs that function as enzymatic domain inhibitors on mutant, over-expressed, or otherwise essential proteins within the cancer cells [188]. 4.) Bone marrow transplantation employs the transfer of hematopoietic stem cells to a recipient with the goal of restoring and repopulating the hematopoietic system entirely or partially [195].

The role of miRNAs in cancer was first established in 2002 when it was discovered that chronic lymphocytic.

Leukemia had downregulated miR-15 and miR-16-1, since then various studies have demonstrated the role of different miRNAs in tumorigenesis, metastasis, and drug resistance [196]. The establishment of the involvement of miRNAs in cancer supports the notion, that certain subsets of miRNAs may be useful as therapeutic agents, as they could target the oncogenic mRNAs [197] and this concept led to the emergence of miRNA-based therapy, as a strategy for cancer treatment.

The development of miRNA-based therapeutics consists of the following main stages, i.e., 1.) Identification of candidate miRNAs for therapy by miRNA profiling of tumor. 2.) Target prediction and validation of candidate miRNA. 3.) Stabilization and encapsulation of candidate miRNA in nano-carriers. 4.) *In-vitro* study of miRNA loaded nano-carriers on cellular cancer model. 5.) Therapeutic candidate miRNA drug testing on animal models. 6.) Clinical trials by assessing the efficacy and toxicity in preclinical studies [198].

Several miRNA-based therapeutics are under clinical trials with a primary goal of treating several lethal diseases, i.e., hepatitis C infection, polycystic kidney disease, cardiac disease, and cancers (including lung cancer, liver cancer, breast cancer, glioblastoma, thyroid cancer, adrenocortical cancer, ovarian cancer and prostate cancer) (Table 6). A detailed list of therapeutics targeting miRNAs in various types of cancer is presented in Table 7. Several research groups have received patents for the discovery of various miRNA molecules as cancer therapeutics (Table 8).

**Table 6:** List of major miRNA-based cancer therapeutics in the developmental phase

Therapeutics	Target miRNA	Cancer	Clinical trial stage
MesomiR-1	miR-16 mimic	Mesothelioma and NSCLC	Phase I (completed)
MRX-34	miR-34 mimic	Various solid tumors	Phase 1
MRG-106	miR-155	Cutaneous T cell lymphoma	Phase I (completed)
miR-10b	miR-10b	Various tumors	Observational
INT-1B3	miR-193a-3p mimic	Advanced solid tumors	Phase I
MesomiR-1	miR-16	Malignant pleural mesothelioma, NSLC	Phase I
Serum miRNA-25	miR-25	Pancreatic cancer	Observational
miR-10	miR-10	Gliomas	Observational

Note: *Adapted from [198,199] and modified.

**Table 7:** A list of therapeutics, targeting various miRNAs in some important types of cancer

Therapeutics	Cancer	Target miRNA	Mechanism of action
Metformin	Pancreatic cancer, Prostate cancer, breast cancer	miR-34a, miR-21, miR-145, miR-101	Targeting glucose metabolism
Pegylated arginine deiminase	Pancreatic cancer	miR-1291	Targeting glutamine metabolism
AZD3965	Breast cancer	miR-342-3	Targeting lactate metabolism: LDHA inhibitors
5-Fluorouracil	Hepatocellular carcinoma,	miR-210, miR-122	Antimetabolite chemotherapeutic agents
Gemcitabine	Pancreatic cancer	miR-218	Inhibits the secretion of HMGB1
Rapamycin	Renal cancer	miR-21	Targeting fatty acid metabolism

Note: *Adapted from [200]and modified.

**Table 8:** List of some recent patents issued for the discovery of miRNA molecules as cancer therapeutics

Inventor	Application	Patent (country)	Applicant
Xin Wu et al.	Cancer stem cell growth inhibitor using miRNA	Japan	Cancer Stem Tech Inc., Osaka, Japan
Amriti R Lulla and Wafik S EL-Deiry	Treatment of tumors with miRNA targeting CDK4/CDK6	US	Institute for Cancer Research and The Research Institute of Fox Chase Cancer Center, Philadelphia, PA, USA
Chenyu Zhang et al.	New Precursor miRNA and Applications in Tumor Therapy	US	Jiangsu Micromedmark Biotech Co., Ltd., Taizhou, China
Marie Wood et al.	miRNA signature expression in cancer	US	University of Vermont and State Agricultural College, Burlington, VT, USA

Note: *Adapted from [201]and modified.

## Mitochondrial Function as a Target in Cancer Therapeutics

Metabolic reprogramming, which promotes macromolecule synthesis, bioenergetics demand, & cellular survival, is a defining feature of malignancies, and mitochondria as a metabolic hub, drive this rewiring via alteration in a variety of processes like glucose utilization, amino acid metabolism, lipid metabolism, etc. [202]. Along with this, Mitochondria is the site for the generation of onco-metabolites, needed for tumor initiation and progression [43]. Thus, exploring the mechanism of mitochondrial reprogramming in each tumor environment could be a possible avenue for the development of the next generation of anticancer drugs [162]. Several research groups have demonstrated the ability of various inhibitors to target the mitochondrial metabolism in cancer therapy and these are summarized in Table 9.

**Table 9:** List of various drugs, targeting the mitochondrial metabolism in cancer therapy

Drug	Mechanism of action	Cancer	Reference
Venetoclax and azacytidine	Suppression of oxidative phosphorylation induces leukemia stem cell death	Acute myeloid leukemia	[203,204]
Dichloroacetic acid	Inhibits the TCA cycle	Liver cancer	[205]
Rapamycin	Inhibits tumor formation	Various cancers	[206,207]
MitoTEMPOL	Antioxidant (reducing ROS)	Various cancers	[208]
IACS-010759	inhibits proliferation and induces apoptosis	Brain tumor	[209]
Gboxin	Complex V inhibitor	Glioblastoma	[210]
Metformin	Complex I inhibitor	Breast cancer, Colon cancer	[211]
Deguelin	Complex I inhibitor	Melanoma	[212]
Rotenone	Complex I inhibitor	Various cancers	[213]
Tigecycline	Mitochondria protein translation inhibitor	Various cancers	[214]
Atovaquone	Inhibits mitochondrial complex III	Non-small cell lung cancer	[215]
Gamitrinib	OXPHOS assembly inhibitor	Various cancers	[216]
Enasidenib	IDH2-mutant inhibitor	Acute myeloid leukemia	[217]

Note: *Adapted from [42,44] and modified.

### MitomiRs in cancer therapeutics

Cancer progression includes mitochondrial pathologies and mitomiRs hold great promise for prognostics and diagnostics in clinical medicine. miRNA mimics and anti-miRs have the potential to be therapeutic solutions for various cancers, as they can be utilized to modulate changed miRNA expression or function.

MRX-34: A potential first-in-class miRNA mimic used for cancer therapeutic is MRX-34, which is a liposomal formulation of miR-34a [218]. miR-34a falls in the category of naturally occurring critical regulator of tumor suppression, that is deleted or expressed at low levels in a variety of tumor types. miR-34a is known as a critical regulator of tumor suppression [219], which suppresses the expression of almost 30 oncogenes via a variety of oncogenic pathways. MRX-34 is currently in phase I trial and is administered intravenously twice a week for 3 weeks. It shows its effect by targeting miR-34a and could be used to treat various types of cancer, i.e., Ovarian cancer, Cervical cancer, HCC, Colon cancer, etc. [201].

MRG-106: The oligonucleotide inhibitor MRG-106 inhibits the miRNA miR-155, which has a close mechanistic relationship to cutaneous t-cell lymphoma [220]. It is used to inhibit miR-155 in patients with cutaneous T-cell lymphoma (CTCL). MRG-106 is developed by miRagentherapeutics and is under Phase-I clinical trials for several cancers, such as CTCL, adult T-cell lymphoma/leukemia, B-cell lymphoma, and chronic lymphocytic leukemia [201].

MRG-110 targets miR-92a which controls blood vessel growth and angiogenesis This molecule is being developed at miRagen Therapeutics and is now undergoing a phase-I clinical trial. [201]. In addition to these, several other mitochondrial microRNA-based anti-cancer drugs, undergoing clinical trials are summarized in Table 10.

**Table 10:** List of various mitochondrial microRNA therapeutics, undergoing clinical trials

Therapeutic molecule	Associated mitomiR	Cancer	Stage of clinical trials
MRG-106	miR-155	Blood cancer	Phase I
MRG-201	miR-29b	Blood cancer fibrosis	Phase II
Cetuximab/FOLFOX	miR-31-3p, miR-31-5p	Colorectal cancer	Phase III
interferon-alpha (IFN alpha)	miR-26	Hepatocellular carcinoma	Phase III
Miravirsen	miR-122	Hepatocellular carcinoma	Phase II
RG-101	miR-122	Hepatocellular carcinoma	On hold
MRX-34	miR-34	Advanced Solid Tumor	Phase I
MRG-110	miR-92a	Angiogenesis	Phase I completed

Note: *Adapted from [134] and modified.

## Conclusion

**Figure 6:**
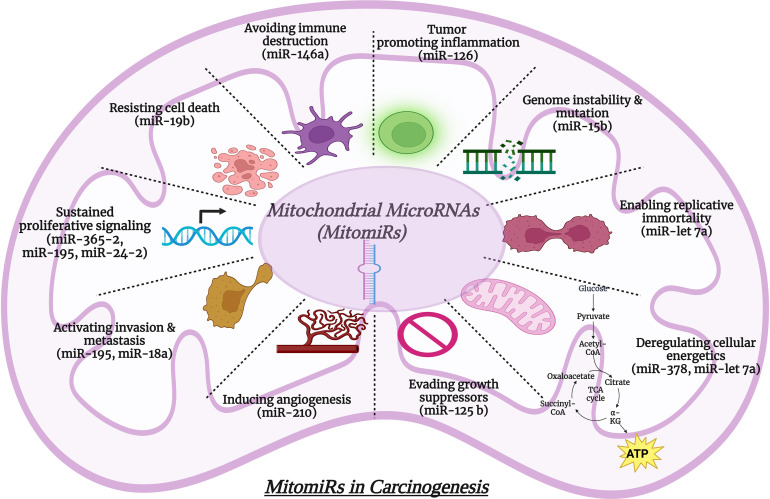
Regulation of various cellular processes by mitomiRs. Created with Biorender.com.

The mitochondrion is a crucial component of cells that is involved in metabolism, signaling cascades, bioenergetics, and cell viability. Dysfunctional mitochondria have been reported to be the root cause of various pathological processes including cancer. Studies in the past decade have brought into light, the role of miRNA in regulating genes and thereby influencing physiological and pathological conditions. Though the general role of miRNA in cellular processes is documented, the importance of the sub-cellular localized domain of miRNA has gradually gained pace in just recent past. MitomiRs are nuclear or mitochondrial-derived miRNAs that are found in mitochondria and play a critical role in controlling metabolism and mitochondrial function. mitomiRs are now reported to be key players in cancer initiation and progression by modulating the hallmarks of cancer. Considering their fascinating intricacy and functional diversity, deciphering the role of mitomirs in carcinogenesis has become an emerging field of research in recent years. In this review, we discussed the hallmarks of cancer, the function of particular mitomirs, their target genes, and functions, and delineated the mechanisms by which mitomiRs modulate mitochondrial metabolism and oncogenic signaling pathways and impact the initiation and progression of cancer. Thus, the less studied aspect of mitomiRs in cancer progression may prove to be a crucial area for future study in order to target cancer cells. Research exploring mitomiRs may aid in cancer diagnosis, prognostic evaluation, and therapeutic targeting. Fig. 6 briefly illustrates the regulation of various cellular processes by mitomiRs.

## Data Availability

Not applicable.
